# TGFβ-activated kinase 1 signaling controls acquisition of the inflammatory fibroblast phenotype and regulates cardiac remodeling after myocardial infarction

**DOI:** 10.21203/rs.3.rs-6122755/v1

**Published:** 2025-03-12

**Authors:** Daniel C. Nguyen, Jonah K. Stephan, Robert E. Brainard, Kenneth R. Brittian, Lianay Gutierrez Luque, Collin K. Wells, Madison S. Taylor, Yania Martinez-Ondaro, Kara R. Gouwens, Danielle T. Little, Nolan Boyd, Richa A. Singhal, Jason Hellmann, Marcin Wysoczynski, Bradford G. Hill

**Affiliations:** 1 Center for Cardiometabolic Science, Christina Lee Brown Envirome Institute, School of Medicine, University of Louisville, Louisville, KY; 2 Department of Physiology, University of Louisville, Louisville, KY; 3 Department of Biochemistry and Molecular Genetics, University of Louisville, Louisville, KY

## Abstract

Organ health and function depend on communication between cell types to coordinate tissue growth and repair. Recent studies have indicated that fibroblasts are critical to this process; however, their role in regulating inflammatory responses to injury have remained ambiguous. Here, we demonstrate that transforming growth factor β-activated kinase 1 (TAK1) is a gatekeeper of the inflammatory cardiac fibroblast phenotype. We find that TAK1 propagates IL-1β and TNF-α signaling in cardiac fibroblasts and coordinates the synthesis and secretion of chemokines as well as inflammatory and pro-resolving lipid mediators. Deletion of TAK1 in fibroblasts decreased immune cell recruitment after MI, which was associated with improved cardiac structural and functional remodeling in male mice. Nevertheless, we found the effects of TAK1 deletion to be sexually dimorphic in nature, providing support to the idea that the protected phenotype of the female sex may be based in disparate immune and inflammatory responses. Moreover, TAK1 signaling controlled the acquisition of novel markers of the inflammatory fibroblast phenotype, having a biological basis in redox stress, chemokine and lipid mediator biosynthesis, metalloproteinase activity, and damage-associated molecular pattern recognition. Collectively, these results further resolve the nature and function of inflammatory cardiac fibroblasts in cardiac responses to injury and identify TAK1 signaling in fibroblasts as a potential target for therapy.

## INTRODUCTION

Inflammation is a highly coordinated response to tissue infection or injury. Although causally linked to fibrosis and organ dysfunction, inflammation is also required for tissue repair. The duplicitous nature of inflammation and its relationship with fibrosis are clearly illustrated in the context of myocardial infarction (MI), in which a burst of inflammatory and fibrogenic programs are temporally initiated to limit excessive injury, clear damaged cardiac tissue, and facilitate replacement fibrosis^1^; however, low-grade inflammation commonly persists after cardiac repair, leading to adverse myocardial remodeling or negatively affecting other processes related to cardiovascular disease risk (e.g., atherosclerotic plaque progression)^1,2^. Recent studies indicate that inflammatory cues from myeloid cells to cardiac fibroblasts are critical to cardiac remodeling and function, with interleukin 1 (IL-1) signaling blockade mitigating fibrosis and heart failure in preclinical models^3,4^ and having promising effects on cardiovascular endpoints and heart failure in humans^5^. Nevertheless, it remains unclear how inflammatory signals influence fibroblast phenotype and function and contribute to cardiac remodeling under conditions of acute and chronic myocardial stress.

After severe tissue injury, such as MI, fibroblasts undergo a transition to an inflammatory phenotype distinct from myofi fibroblasts^6^. Inflammatory fibroblasts have been suggested to play critical roles in paracrine and autocrine signaling, to coordinate immune cell recruitment and activation, and to reduce adverse cardiac remodeling after MI^7,8^; however, it remains unclear how fibroblasts acquire this distinct inflammatory phenotype and how they influence cardiac remodeling. Among the pathways that could propagate inflammatory and profibrotic signaling, TGF-β activated kinase 1 (TAK1) appears to be a conspicuous candidate. TAK1 is a 579 amino acid MAPKK kinase that is constitutively expressed in most cell types, with the capacity to coordinate downstream mediators involved in inflammatory responses^9,10^. Although initially identified as a TGF-β-responsive enzyme, studies in different cell models have reported that TAK1 coordinates signaling from IL1 receptors, toll-like receptors (TLRs), and tumor necrosis factor (TNF) receptors, stimulating downstream inflammatory pathways including NF-κB, JNK, and p38^11^. Despite TAK1 being uniquely positioned to integrate distinct signals that are critical in cardiac repair, its role in regulating cardiac fibroblast biology and fibroblast-mediated cardiac remodeling and repair remain unexplored. Therefore, in this study, we examined the role of TAK1 in acquisition of the inflammatory fibroblast phenotype and in modulating post-MI immune cell recruitment and cardiac remodeling. We report that TAK1 is a gatekeeper of the inflammatory cardiac fibroblast phenotype that coordinates inflammatory signaling, chemokine and (anti)inflammatory lipid mediator production, immune cell recruitment, and myocardial remodeling after MI. Collectively, these findings identify TAK1 as a novel signaling mechanism that underlies the differentiation of inflammatory cardiac fibroblasts and post-MI remodeling.

## RESULTS

### TAK1 regulates inflammatory signaling, protein synthesis, and protein secretion in cardiac fibroblasts.

**Figure 1:**
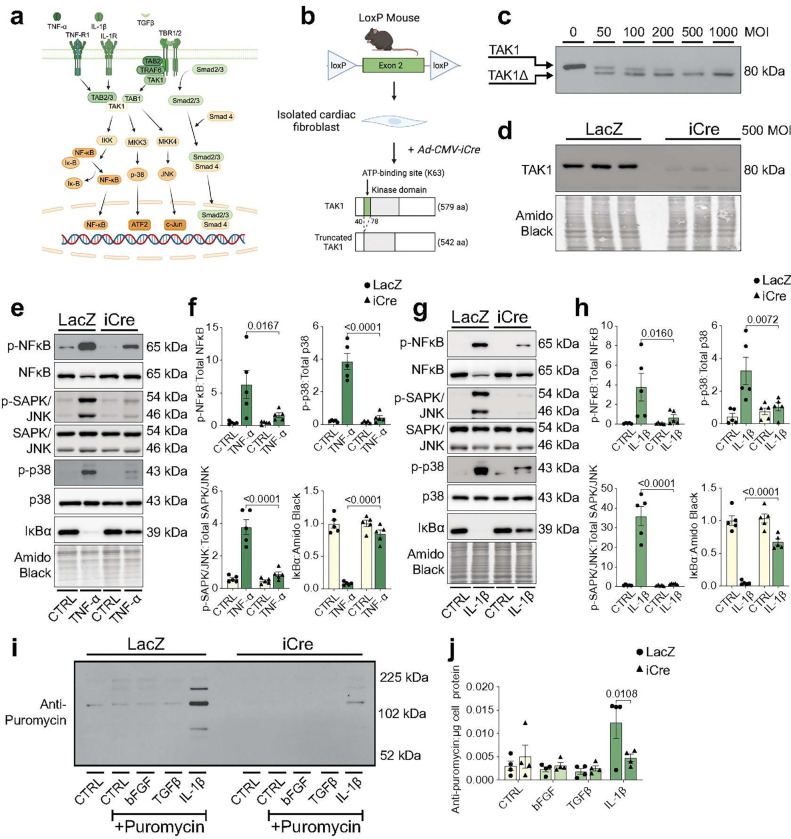
TAK1 propagates inflammatory signaling and regulates the synthesis of secreted proteins in cardiac fibroblasts. **a,** Illustration of TAK1 signaling. **b,** Schematic of TAK1 conditional allele with LoxP sites flanking exon 2. Cre recombination yields a truncated TAK1 product. **c, d,** Validation of Cre-mediated TAK1 truncation (TAK1Δ) in isolated TAK1^*fl/fl*^ cardiac fibroblasts 3 (**c**) and 7 (**d**) days after adenovirus (Ad-CMV-iCre) treatment. **e–h,** Representative immunoblots and densitometry of inflammatory and stress signaling proteins in control (Ad-CMV-LacZ) or iCre-pretreated TAK1^*fl/fl*^ cardiac fibroblasts. fibroblasts were treated without or with TNF-α (**e, f**) or IL-1β (**g, h**) for 30 min prior to cell lysis and collection. **i, j,** Evaluation of puromycin-tagged proteins in conditioned medium from control or TAK1 deficient fibroblasts following 48 h of ligand stimulation. Data are mean ± SEM. For **d**–**j**, n = 3–5 biological replicates per group. Statistical procedures: (**f,h,j**) two-way ANOVA with Šidák’s post-hoc. Experiments and blots were processed in parallel and normalized to an anchor protein. Schematics in **a**,**b** were created using BioRender (https://biorender.com).

Recent studies identified ligands such as IL-1β and TGFβ to be among the strongest signals received by fibroblasts after MI^3,4^. In the context of cardiovascular diseases, IL-1β and its associated signaling responses are of interest, given the potential efficacy of IL-1β blockade (reviewed in^12^). Moreover, other ligands involved in inflammation and cardiac repair, such as TNF-α and TGF-β, respectively, are also elevated after MI and play important roles in myocardial remodeling and fibrosis^1^.Collectively, these ligands are capable of triggering inflammatory (NF-κB), stress kinase (p38, JNK), or Smad pathway activation (Fig. 1a). To determine whether ligands known to be elevated after MI activate downstream inflammatory and stress kinase signaling in cardiac fibroblasts, we first treated fibroblasts isolated from adult, murine hearts with IL-1β, TNF-α, or TGFβ and examined inflammatory and stress kinase signaling. Treatment with IL-1β or TNF-α caused rapid (within 15 min) and robust activation of the NFκB pathway, as exemplified by increased p-NFκB abundance and IκBα degradation. Moreover, IL-1β treatment activated both SAPK/JNK and p38 to a greater extent than the other tested ligands (**Supplementary Fig. 1a**).

Inflammatory and stress kinase signaling may be important in the context of tissue injury, given that IL-1β signaling attenuates TGFβ-induced myofibroblast differentiation and regulates the synthesis and secretion of cytokines and chemokines^8^. Indeed, we found that IL-1β attenuated TGFβ-induced periostin and α-smooth muscle actin (α-SMA) abundance, but did not affect collagen upregulation (**Supplementary Fig. 1b–1e**). To determine how IL-1β influences protein synthesis and secretion, we leveraged the SUnSET assay^13^ to measure the secretion and relative abundance of newly synthesized proteins in the culture medium. Cardiac fibroblasts were serum-starved (control) or treated with basic fibroblast growth factor (bFGF), TGFβ, or IL-1β for up to 48 h, followed by a 30 min pulse of low-dose puromycin—a structural analog of tyrosyl-tRNA that is incorporated into elongating peptide chains. Medium from IL-1β-treated cells showed markedly higher levels of puromycin-labeled proteins than any other tested ligand, indicating that IL-1β signaling augments protein translation velocity and secretion (**Supplementary Fig. 1f, 1g**). Although both IL-1β and TGFβ promoted secretion of collagen into the medium (**Supplementary Fig. 1f, 1h**), only TGFβ increased periostin (**Supplementary Fig. 1f, 1i**), indicating that the puromycin-labeled proteins from IL-1β-stimulated fibroblasts likely consist of proteins other than fundamental components of the extracellular matrix.

We next determined the extent to which proinflammatory and fibrotic signaling is propagated through TAK1, which appears to be a signaling nexus with cell type-specific functions^14^ (Fig. 1a). To determine the role of TAK1 in regulating inflammatory signaling in cardiac fibroblasts, we used a murine TAK1^*fl/fl*^ model, which possesses LoxP sites flanking exon 2 of the TAK1 (*Map3k7*) gene^15^ (Fig. 1b). Exon 2 encodes the ATP-binding site, necessary for TAK1 phosphorylation and activation; hence, Cre-mediated recombination in TAK1^*fl/fl*^ cardiac fibroblasts should lead to a truncated TAK1 product (TAK1Δ) with abolished kinase activity. Indeed, adenoviral expression of Cre recombinase (Ad-CMV-iCre) led to the appearance of TAK1Δ within 3 d of genetic recombination (Fig. 1c); further culture for 4 additional days led to disappearance of the TAK1Δ product, suggesting that TAK1Δ is unstable and ultimately degraded (Fig. 1d). Notably, TAK1 deletion did not affect cell number, suggesting that it likely does not influence cell proliferation or viability in unstimulated cells (**Supplementary Fig. 2a**). Genetic deletion of TAK1 nearly abolished both TNFα- and IL-1β-mediated activation of the NFκB, p38, and SAPK/JNK signaling pathways (Fig. 1e–1h); however, TAK1 deletion did not affect TGFβ-induced activation of SMAD3 or p38 (**Supplementary Fig. 2b, 2c**), suggesting that, in cardiac fibroblasts, TAK1 propagates inflammatory, but not SMAD, signaling. Interestingly, TAK1 deletion also abrogated puromycin labeling of secreted proteins observed with IL-1β treatment, suggesting that it controls inflammatory ligand-initiated translation and secretion of proteins from cardiac fibroblasts (Fig. 1i, 1j).

### Fibroblast-specific TAK1 deletion influences cardiac remodeling after myocardial infarction, but not pressure overload.

To delineate the *in vivo* role of fibroblast TAK1 signaling in the heart, we bred TAK1^*fl/fl*^ mice to mice expressing tamoxifen-inducible Cre under the control of the Col1a2 promoter. We used the Col1a2 Cre approach because, in genetic fate mapping studies, Col1a2-Cre^ERT^ transgenic mice express Cre predominantly in fibroblasts, with Cre expressed in cells harboring fibroblast markers (e.g., *Vim*, *Ddr2*), but not expressed in endothelial cells or cardiomyocytes^16^. We validated TAK1 knockdown in fibroblasts by assessing TAK1 expression in isolated cardiac fibroblasts from Cre^−^ and Cre^+^ mice administered tamoxifen, which demonstrated robust and persistent deletion of TAK1 (**Supplementary Fig. 3a–3c**). Echocardiographic assessments showed that deletion of TAK1 in fibroblasts for up to 16 weeks following tamoxifen administration has no effect on cardiac structure or function in the unstressed condition (**Supplementary Tables 1, 2**).

To delineate whether fibroblast TAK1 signaling is important during chronic cardiac stress, we examined the effect of TAK1 deletion on cardiac structure and function in the pressure overloaded heart. Following tamoxifen administration, Cre^−^ and Cre^+^ TAK1^*fl/fl*^ mice were subjected to transverse aortic constriction (TAC), with serial echocardiography performed prior to surgery (baseline; BSL) and at 4, 8, and 12 weeks post-TAC (**Supplementary Fig. 4a**). Gravimetric data indicated no changes in body mass 12 weeks post-TAC and evaluation of heart weight (HW) normalized to tibia length (TL) indicated similar heart mass between groups (**Supplementary Fig. 4b, Supplementary Tables 3, 4**). Serial assessments of cardiac function illustrated progressive development of heart failure, with both Cre^−^ and Cre^+^ mice demonstrating ventricular dilatation and worsening ejection fraction over the 12-week period, with no significant differences in any parameter between groups (**Supplementary Table 3, 4**). Similarly, cardiac collagen content measured via hydroxyproline assay 12 weeks post-TAC revealed no differences between Cre^−^ and Cre^+^ mice (**Supplementary Fig. 4c**). At the conclusion of the study, we conducted pressure-volume loop studies in a subset of the TAC mice to assess cardiac function in detail; however, indicators of cardiac compliance (EDPVR) and contractility (ESPVR) showed no significant difference between Cre^−^ and Cre^+^ mice (**Supplementary Table 5**). Collectively, these results suggest that fibroblast TAK1 signaling does not influence pathological remodeling caused by pressure overload.

**Figure 2:**
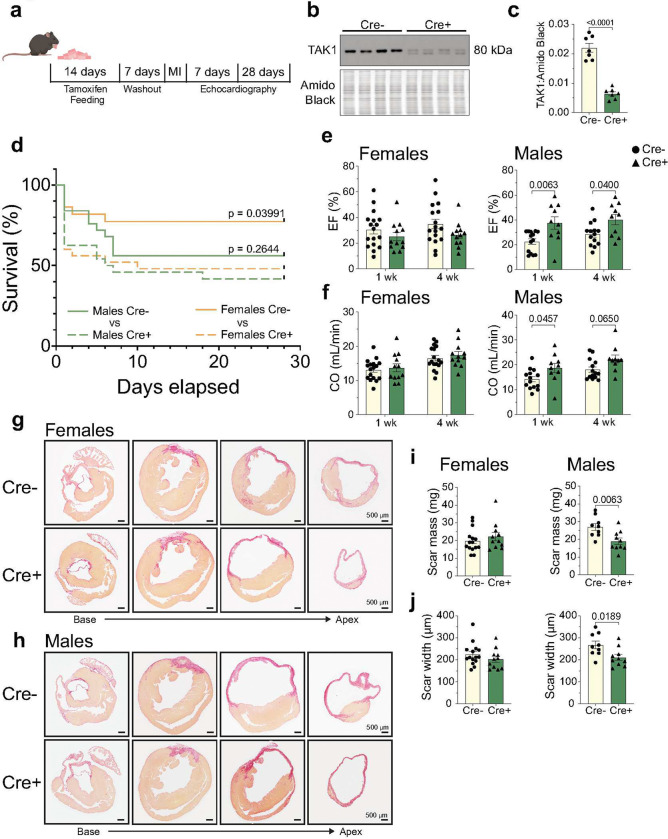
Fibroblast TAK1 deletion attenuates adverse structural and functional cardiac remodeling following myocardial infarction in male mice. **a,** Experimental scheme of mice subjected to MI injury with echocardiography acquisition at 7 and 28 d post-MI. **b,c,** Representative immunoblot (**b**) and densitometric quantification (**c**) of TAK1 knockdown from cardiac fibroblasts isolated from tamoxifen-treated mice. Proteins were normalized to amido black signal from stained membranes. n = 3–4 independent biological replicates per group, with males and females pooled. **d,** Kaplan-Meier survival curves for Cre^−^ and Cre^+^ TAK1^*fl/fl*^ male (n = 49; 25 Cre^−^, 24 Cre^+^) and female (n = 47; 22 Cre^−^, 25 Cre^+^) mice following MI. **e,f,** Echocardiographic quantification of ejection fraction (EF; **e**) and cardiac output (CO; **f**) at 1 and 4 wk post-MI for female and male mice. n = 10–17 biological replicates per group. **g–j,** Representative Picosirius red staining of transverse myocardial sections (**g, h**) used for quantification of scar mass (**i**) and width (**j**) from infarcted hearts. n = 9–15 biological replicates per group. Data are mean ± SEM. Statistical procedures: Normality was assessed via Shapiro-Wilk test. (**c**) Parametric unpaired student’s t-test. (**d**) log-rank (Mantel-Cox) test. (**e, f**) two-way ANOVA with Šidák’s post-hoc. (**i, j**) Parametric unpaired student’s t-test or non-parametric Mann-Whitney U test were used as appropriate. Schematics in **a** were created using BioRender (https://biorender.com).

To determine the extent to which TAK1 signaling in fibroblasts influences cardiac remodeling after MI, Cre^−^ and Cre^+^ TAK1^*fl/fl*^ mice were administered tamoxifen prior to permanent coronary ligation (Fig. 2a). In a subset of mice, sufficient recombination was ensured by isolating cardiac fibroblasts and measuring TAK1 abundance (Fig. 2b, 2c). Serial echocardiography was then performed at 1- and 4-weeks post-MI. As expected from this severe MI model^17^, we observed ~50% mortality in male mice; however, there was no significant difference in survival between Cre^−^ and Cre^+^ males (Fig. 2d). In contrast, female Cre^+^ mice showed higher mortality (~50%) than their Cre^−^ counterparts (~20%), expunging the protective attributes of the female sex. Body mass was slightly diminished in male Cre^+^ mice, with no significant changes in heart weight or pulmonary congestion in either sex (**Supplementary Table 6**). Interestingly, ejection fraction and cardiac output were significantly improved in TAK1-deficient males, but were unchanged in TAK1-deficient females (Fig. 2e, 2f). Similarly, assessment of collagen burden measured via picrosirius red staining showed decreased fibrotic mass and transmural scar width in TAK1-deficient male mice, with no change in fibrosis in female mice (Fig. 2g–2j). These findings indicate that fibroblast TAK1 has sexually dimorphic effects in the context of MI, with TAK1 deletion improving cardiac function in male mice after MI, but increasing mortality in female mice.

### Fibroblast TAK1 activity regulates immune cell abundance in the heart following MI.

**Figure 3:**
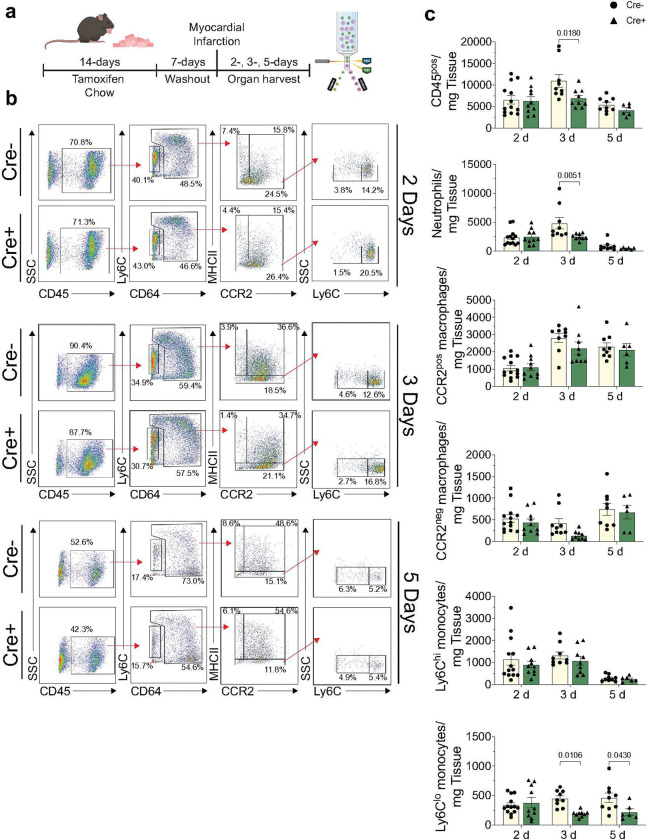
Fibroblast TAK1 deletion diminishes the abundance of immune cells in the heart following MI. **a,** Schematic of study design assessing cardiac immune cell abundances 2, 3, and 5 d post-MI. **b,** Flow cytometric gating strategy for identification and quantification of immune cell populations. **c,** Populations were quantified and normalized to wet tissue weight. n = 6–13 biological replicates per group. Data are mean ± SEM. Statistical procedures: two-way ANOVA with Šidák’s post-hoc. Schematics in **a** were created using BioRender (https://biorender.com).

Based on our observation that TAK1 controls inflammatory and stress signaling pathways that could regulate chemoattractant secretion, we assessed the significance of inflammatory cardiac fibroblasts on immune cell abundance in the heart after MI. Following tamoxifen administration to Cre^−^ and Cre^+^ TAK1^*fl/fl*^ mice, the mice underwent permanent ligation surgery and hearts were harvested at 2, 3, and 5 d post-MI to assess immune cell composition and abundance (Fig. 3a). Flow cytometric strategies that focus on monocyte and macrophage subtypes and that delineate neutrophils using exclusion criteria^18^ were used to enumerate immune cells in the infarcted heart (Fig. 3b). As shown in Fig. 3c, the abundance of CD45^pos^ leukocytes was significantly reduced 3 d post-MI in male Cre^+^ mice. Further stratification of distinct immune cell populations demonstrated a reduction in neutrophils and Ly6C^lo^ monocytes post-MI in Cre^+^ hearts (Fig. 3c). Evaluation of CD45^pos^ leukocyte and monocyte populations in bone marrow and the peripheral circulation showed generally higher levels in Cre^+^ mice despite being lowered within the heart, suggesting that immune cell infiltration into the post-MI heart is blunted (**Supplementary Fig. 5**). These results indicate that TAK1 signaling in cardiac fibroblasts influences the abundance of immune cells in the heart after MI.

### TAK1 regulates chemokine/cytokine and lipid mediator secretion in a diametrically opposite manner:

**Figure 4:**
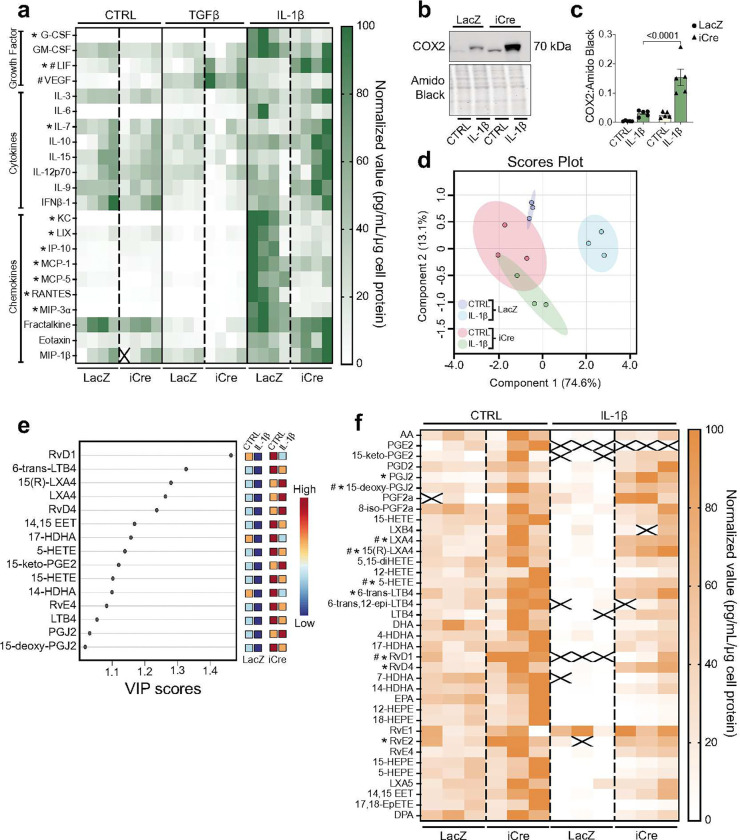
The IL-1β-mediated fibroblast secretome is coordinated via TAK1 signaling. Isolated cardiac fibroblasts transduced with LacZ or iCre adenovirus were treated with TGFβ or IL-1β for 48 h prior to conditioned medium collection. **a,** Heat map illustrating secreted proteins measured using quantitative cytokine/chemokine assay array (MD44; Evetechnologies). X = outside of standard curve, n = 4 male biological replicates per group. **b, c,** Representative immunoblot (**b**) and densitometry quantification (**c**) of cyclooxygenase 2 (COX2) abundance from TAK1^*fl/fl*^ and TAK1Δ fibroblasts following 48 h of IL-1β stimulation. Data are mean ± SEM. Proteins were normalized to amido black signal from stained membranes. n = 5 male biological replicates per group. **d–f,** Conditioned medium was collected for LC-MS/MS analysis of secreted lipid mediators: PLS-DA plot (**d**), VIP score plot (**e**), and heatmap (**f**). n = 3 male biological replicates per group. Statistical procedures: (**a, c, f**) two-way ANOVA with Šidák’s post-hoc test; (**a**) *^#^ p<0.05, *IL-1β (LacZ vs iCre), ^#^TGFβ (LacZ vs iCre); (**f**) *^#^ p<0.05, *IL-1β (LacZ vs iCre), ^#^CTRL (LacZ vs iCre).

Because IL-1β converted cardiac fibroblasts to a highly secretory phenotype (Fig. 1i; **Supplementary Fig. 1f, 1g**) and given data demonstrating reduced recruitment of immune cells (Fig. 3c), we performed quantitative chemokine and cytokine arrays to determine which secreted proteins are controlled by TAK1 signaling. TAK1^*fl/fl*^ fibroblasts transduced with control (LacZ) or iCre adenovirus were exposed to either TGFβ or IL-1β for 48 h prior to conditioned medium collection. Although chemokine/cytokine measurements showed that TAK1 deletion had no effect on cytokine or chemokine abundance in the medium of serum-starved cells, TGFβ increased VEGF abundance, which was further elevated with iCre treatment (Fig. 4a). After IL-1β stimulation, we observed robust secretion of growth factors (G-CSF, GM-CSF) and chemokines (KC, LIX, IP-10, MCP-1, MCP-5, Rantes, MIP-3α, Eotaxin) known to play key roles in immune cell recruitment and activation. Deletion of TAK1 inhibited nearly all IL-1β-induced growth factors and chemokines, with only leukemia inhibitor factor (LIF) increasing with TAK1 deletion (Fig. 4a). These findings indicate that TAK1 is a positive regulator of pro-inflammatory protein chemoattractant synthesis and secretion.

In addition to secreted proteins, lipid mediators are crucial in both initiating and quelling inflammation^19^. Although a cascade of enzymes is typically required for lipid mediator synthesis, we first examined how TAK1 influences the abundance of cyclooxygenase 2 (COX2), which is involved in synthesis of both inflammatory and proresolving lipids^20^. Interestingly, IL-1β treatment increased COX2 abundance in TAK1-replete cells; however, IL-1β treatment super-induced COX2 in the context of TAK1 deficiency (Fig. 4b, 4c). Analysis of LC/MS lipidomics data via partial least squares-discriminant analysis showed distinct group separation in TAK1-replete cells treated with IL-1β, with group separation abolished in the context of TAK1 deficiency (Fig. 4d). To determine the variables driving group separation, we performed variable importance in projection (VIP) score analysis, which highlighted proresolving and inflammatory lipid mediators (e.g., resolvins, lipoxins, prostaglandins, leukotrienes) to be principal factors driving differences (Fig. 4e). Interestingly, IL-1β diminished levels of not only lipid mediator products but also their substrates (i.e. arachidonic acid, docosahexaenoic acid, eicosapentaenoic acid), indicating that IL-1β-mediated signaling diminishes lipid mediator synthesis, at least in part, by controlling substrate levels (Fig. 4f). Deletion of TAK1 not only rescued the attenuation of lipid mediators caused by inflammatory stimuli, but also augmented production of lipid mediators, even in the absence of IL-1β stimulation. These results indicate that TAK1 regulates chemokines and lipid mediators in a diametrically opposite fashion, where TAK1 is a negative regulator of lipid mediator secretion but a positive regulator of chemokine/cytokine secretion, insinuating that its activation state regulates distinct processes in cardiac inflammation and resolution.

### TAK1 is a gatekeeper of the inflammatory cardiac fibroblast phenotype.

**Figure 5:**
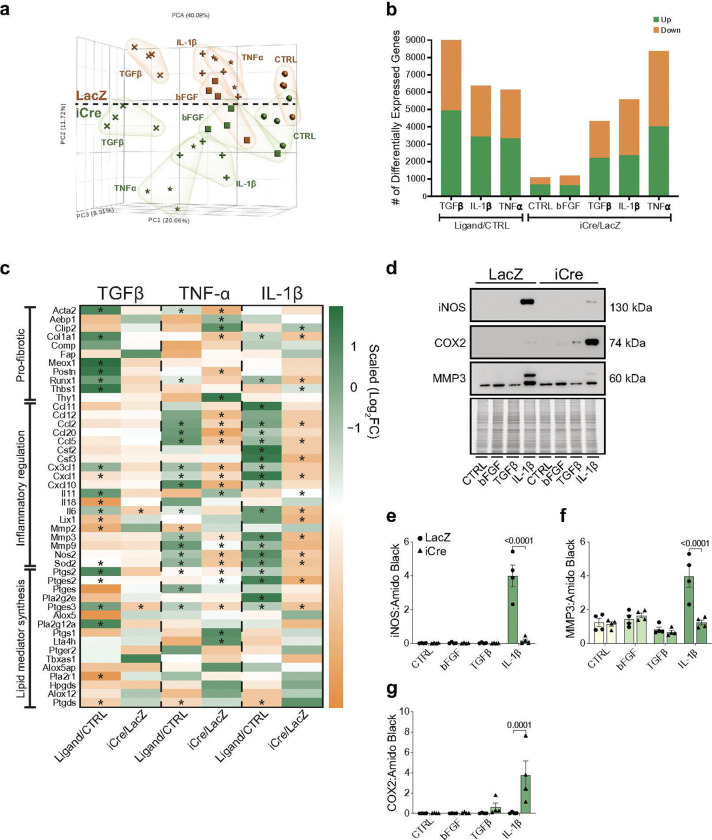
TAK1 controls acquisition of an inflammatory fibroblast phenotype. **a,** Principal component analysis of gene expression from LacZ or iCre transduced cells following 24 h of ligand stimulation. **b,** Plot indicating the number of differentially expressed genes across comparison groups (log_2_FC > 0; q-value < 0.05). **c,** Heat map visualization of the log_2_FC values for genes associated with fibrotic processes, inflammatory regulation, and lipid mediator synthesis. *q < 0.01. **d–g,** Representative immunoblots (**d**) and densitometric quantification (**e–g**) of iNOS, COX2, and MMP3 levels from TAK1^*fl/fl*^ and TAK1Δ fibroblasts following 48 h of ligand stimulation. Data are mean ± SEM. Proteins were normalized to amido black signal from stained membranes. Experiments and blots were processed in parallel and normalized to an anchor protein. For **a–g**, n = 4 male biological replicates per group. Statistical procedures: (**e–g**) two-way ANOVA with Šidák’s post-hoc test.

To further elucidate the nature by which TAK1 regulates the cardiac fibroblast phenotype, we performed bulk RNA sequencing of fibroblasts stimulated with bFGF, TNF-α, TGFβ, or IL-1β for 24 h, with serum-starved cells serving as a general control. The evaluation of gene markers in the serum-starved control group indicates that the isolated cells exhibit a fibroblast expression profile (**Supplementary Table 7**). Principal component analysis (PCA) revealed distinct cluster separation based on ligand treatment relative to the general control, with TAK1 deletion strongly modulating gene variance among TNFα and IL-1β stimulated cells (Fig. 5a). Indeed, analysis of DEGs showed that deletion of TAK1 had larger effects on fibroblast gene expression in the context of TNF-α and IL-1β treatment, with relatively minimal TAK1-dependent changes were apparent within the CTRL and bFGF groups. Although compared with inflammatory ligands, fewer TGFβ-regulated DEGs were under TAK1 control, approximately 4000 genes were influenced by TAK1 deletion in TGFβ-treated cells (Fig. 5b). Nevertheless, myofibroblast-related pro-fibrotic genes were not significantly changed by TAK1 deletion (Fig. 5c), which is in alignment with data showing that TAK1 deletion influences neither Smad3 signaling (**Supplementary Fig. 2b, 2c**) nor acquisition of markers of the myofibroblast phenotype or ECM components such as hyaluronan^21^ (**Supplementary Fig. 6**). Differential gene expression analysis indicated that both TNFα and IL-1β regulate similar gene sets, including notable changes in (anti)oxidant genes (e.g., *Nos2*, *Sod2*), chemokines/cytokines (e.g., *Cxcl* and *Ccl* gene families), chemotactic growth factors (e.g., *Csf2*), and matrix metalloproteinases (e.g., *Mmp* gene family), which were under the control of TAK1 (Fig. 5c). We also found that genes regulating lipid mediators were influenced by inflammatory stimuli, with many under the control of TAK1 signaling. Collectively, these results identify TAK1 as a vital enzyme responsible for conveying extracellular cues to trigger an inflammatory transcriptional profile.

Guided by our RNA-seq data, we confirmed changes in several potential indicators of the inflammatory cardiac fibroblast phenotype. Given the robust upregulation of *Nos2* (iNOS), *Ptgs2* (Cox2), and *Mmp3*, we first confirmed the extent to which inflammatory stimuli such as IL-1β influences their protein abundance. Consistent with transcript patterns, IL-1β robustly increased iNOS and MMP3 protein levels, which were abrogated in TAK1-deficient fibroblasts (Fig. 5d–5g). Similar to findings in Fig. 4b, COX2 protein levels were markedly elevated in TAK1-deficient fibroblasts treated with IL-1β (Fig. 5d, 5g), indicating that TAK1 likely controls the translational efficiency of *Ptgs2* or proteolysis of COX2 to regulate its protein abundance. Indeed, interrogation of RNA sequencing data showed that processes related to proteasomal processing were markedly impacted by TAK1 deletion (**Supplementary Fig. 7**). Volcano plot analysis also showed that toll-like receptor 2 (*Tlr2*) was the most highly upregulated member of the TLR family in response to IL-1β and that TAK1 regulated not only *Tlr2*, but also *Tlr3*, *Tlr4*, and *Tlr5* (**Supplementary Fig. 8a–c**). Furthermore, flow cytometric analysis of TLR2 surface abundance demonstrated that IL-1β increases surface expression of TLR2 (**Supplementary Fig. 8d, 8e**). Collectively, these data show that TAK1 is a gatekeeper of inflammatory cardiac fibroblasts and that iNOS, MMP3, and TLR2 may be useful markers of the inflammatory cardiac fibroblast phenotype.

## DISCUSSION

Tissue repair and remodeling depend on communication between different cell types to coordinate removal of cellular detritus, synthesis of extracellular matrix, and restoration of tissue. Although it is understood that fibroblasts are critical to this process, it has remained unclear how inflammatory processes in fibroblasts coordinate cellular communication with immune cells to respond to tissue injury. We show in the context of myocardial injury that TAK1 signaling in fibroblasts is a gatekeeper of the inflammatory cardiac fibroblast phenotype. Our findings indicate that deletion of TAK1 in fibroblasts improves cardiac structural and functional remodeling after MI, which is associated with diminished post-MI abundance of immune cells and lower levels of resident macrophages. Interestingly, the effects of TAK1 deletion on post-MI remodeling are sexually dimorphic, which provides support to the idea that the protected phenotype of the female sex may have a basis in disparate immune and inflammatory responses. Moreover, TAK1 was found to regulate the synthesis and secretion of chemokines and (anti)inflammatory lipid mediators in a diametrically opposite manner. Identification of TAK1 as the critical enzyme propagating inflammatory cues provided the opportunity to further define markers of the inflammatory cardiac phenotype. Collectively, these findings clarify the nature and function of inflammatory cardiac fibroblasts in cardiac responses to injury and further resolve working models of how cardiac repair proceeds after MI.

Inflammatory signaling has been a focus of numerous therapeutic efforts to combat heart disease. Several studies demonstrate that blunting IL-1 signaling or its downstream targets (e.g, NFκB) attenuates cardiac dysfunction^3,4,8,22^. Nevertheless, it remains unclear how inflammatory signaling in cardiac fibroblasts is coordinated and how it contributes to cardiac responses to stress or injury. Our investigations of fibroblast-resident TAK1 have uncovered its role as a central hub for integrating diverse inflammatory signals to drive inflammatory cardiac fibroblast differentiation and function. Using both acute and chronic models of cardiac injury, we found that TAK1 signaling in fibroblasts is critical for cardiac remodeling after acute MI, whereas it has no effect on pressure overload-induced cardiac remodeling. These findings suggest that fibroblast TAK1 signaling may be critical for myocardial injuries that are followed by robust inflammatory responses, but that it does not affect chronic responses to cardiac stress, which are typically associated with relatively lower levels of inflammation. Deletion of TAK1 in fibroblasts resulted in improved functional outcomes as early as one-week post-MI in male mice, with sustained improvement throughout the 4-week study period. Additionally, TAK1 deletion in fibroblasts attenuated fibrosis after MI. Interestingly, TAK1 deletion had salutary effects only in male mice, with female mice showing no improvement in cardiac function or fibrotic burden; rather, TAK1 deletion in female mice worsened survival to levels observed in male mice. These findings place TAK1 signaling in fibroblasts as a critical, sexually dimorphic regulator of post-MI remodeling and survival.

The fact that TAK1 propagates IL-1β and TNFα signaling in cardiac fibroblasts, but does not seem to affect canonical TGFβ signaling, suggests that its primary function is to regulate acquisition of properties associated with inflammation. An inflammatory fibroblast phenotype would be expected to exhibit a capacity to rapidly synthesize and secrete cytokines, chemokines, and growth factors; to sense molecular patterns educed by tissue injury (e.g., DAMPs, PAMPs); and to secrete proteases that aid in extracellular matrix remodeling^6^. Our findings demonstrate that TAK1 is essential for acquiring these unique properties, exerting robust control over the synthesis and secretion of chemokines, (anti)inflammatory lipid mediators, and matrix metalloproteinases (e.g., MMP3). The synthetic and secretory capacity of fibroblasts exposed to IL-1β was especially robust, with translation velocity assays and cytokine/chemokine arrays indicating that TAK1 signaling is important for the rapid secretion of several growth factors (G-CSF, GM-CSF) and chemokines (e.g., MCP1, MCP5, LIX, KC, MIP3α) that are known to trigger immune cell recruitment. Indeed, *in vivo* analysis of immune cell populations in the post-infarcted heart showed that TAK1 deletion decreased the abundance of CD45^pos^ cells, including neutrophils and Ly6C^lo^ monocytes. Although canonical TGFβ-SMAD3 signaling was not affected by TAK1 deletion, TGFβ-induced VEGF secretion was higher in TAK1-deficient fibroblasts, suggesting that it may nevertheless exert some control over non-canonical TGFβ signaling and thereby control not only the inflammatory properties of fibroblasts, but their proangiogenic properties as well.

TAK1 deficiency also mitigated IL-1β-induced upregulation of toll-like receptors (TLRs), implying that another function of TAK1 is to regulate DAMP sensing. Upon ischemic injury, necrotic cardiomyocytes and damaged tissue release DAMPs, which interact directly with pattern recognition receptors such as TLRs to modulate cell function^1^. We suggest that TLRs along with other enzymes unique to the inflammatory fibroblast phenotype likely coordinate and refine tissue remodeling. In particular, the marked upregulation of iNOS would likely lead to local nitrosative or nitrative stress, which could indirectly trigger TLR activation. Peroxynitrite, a prominent product of iNOS, is known to degrade glycosaminoglycans such as hyaluronan to smaller fragments^23^, which act as endogenous danger signals that activate TLRs^24–27^. Thus, the integrated transcriptional landscape triggered by TAK1 signaling likely acts to coordinate and fine tune tissue inflammatory responses, matrix breakdown, and immune cell recruitment to help clear debris and propagate tissue repair processes. These findings appear to warrant further studies of how TLR signaling in inflammatory cardiac fibroblasts contributes to tissue repair and remodeling.

An unexpected finding of our study is that TAK1 signaling, while essential for chemokine synthesis, represses the production of a wide range of lipid mediators, which growing evidence highlights are pivotal in orchestrating inflammatory and pro-resolving responses following MI^28^. Interestingly, IL-1β decreased the abundance of not only final products in lipid mediator synthesis pathways (e.g., prostaglandins, lipoxins, resolvins), but also their initial substrates (e.g., arachidonic acid, eicosapentaenoic acid, docosahexaenoic acid). The fact that TAK1 deletion completely reverted these phenomena suggests that TAK1 signaling exerts strong control of lipid mediator synthesis. These results help form a model in which TAK1 dictates cadence over inflammatory and resolution programs, wherein inflammatory stimuli and associated TAK1 signaling in fibroblasts initially augment production of chemokines to recruit immune cells to the heart and that, once the severe inflammatory state subsides, subsequent loss of TAK1 signaling allows for the production of lipid mediators to help resolve residual inflammation. Nevertheless, there remains much to learn about the mechanisms by which TAK1 signaling controls lipid mediator synthesis. Hints are provided by our transcriptomics data, which show that TAK1 controls the abundance of phospholipase transcripts (**Supplementary Fig. 7**). Moreover, it is likely that the stark increase in COX2 abundance in TAK1-deficient cells stimulated with inflammatory ligands also plays a role in lipid mediator synthesis and could be involved in lipid mediator “class-switching”, which has been proposed to facilitate both inflammation and resolution^29^. Although it remains unclear how TAK1 controls COX2 protein levels, COX2 is known to be tightly regulated at both the level of transcript stability and translational efficiency^30^.

The collective findings of this study help to further refine working models of how fibroblasts contribute to post-MI remodeling. Recent studies report that IL-1β is primarily secreted by Cx3cr1^+^ or CCR2^+^ immune cells, which drive the emergence of distinct cardiac fibroblast subpopulations^3,4^. Our findings develop on the concept that fibroblasts function as a second-tier step in regulating the abundance of immune cells, and that their acquisition of an inflammatory fibroblast phenotype could help coordinate removal of extant debris, the synthesis of extracellular matrix, and the resolution of inflammation. The fact that TAK1 regulates chemokine and lipid mediator synthesis in a diametric fashion suggests that inflammatory fibroblasts could alternately regulate immune cell recruitment and function to resolve inflammation after adequate wound healing has occurred. Nevertheless, several questions remain to be addressed. For example, Amrute et al. showed that IL-1β contributes to the emergence of a fibroblast population characterized by elevated periostin expression^3^; however, our study, as well as others^8^, indicate that IL-1β suppresses TGFβ-induced increases in periostin (and α-SMA). It is likely that, in addition to inflammatory stimuli such a IL-1β and TNFα, a panoply of paracrine mediators contributes to the ultimate phenotype that fibroblasts assume in the post-infarcted heart and that, like macrophages^31^, fibroblasts can assume a spectrum of unique phenotypes and state spaces^32^. Another interesting but unresolved question relates to the strong sexual dimorphism observed with fibroblast TAK1 deletion. That TAK1 deficiency in female mice did not affect fibrosis or cardiac function, and actually increased mortality, appears to suggest that a principal difference in post-infarct remodeling relates to differences in the function of inflammatory fibroblasts between the sexes. Nonetheless, the findings here place TAK1 as a critical signaling conduit that regulates the inflammatory cardiac fibroblast phenotype and cardiac repair after MI.

## METHODS

### Primary Cardiac Fibroblast Isolation and Culture:

Cardiac fibroblasts were isolated from adult, naïve, C57BL/6J wild type (Jackson Laboratory) and TAK1^*fl/fl*^ (gifted by Ashok Kumar^33^) mice. Briefly, after euthanasia, the hearts were excised, and the aorta was cannulated using a 23-gauge needle. Ice-cold PBS was perfused through the aorta, and any excess aorta and adipose tissue were carefully excised from the heart before mincing it with a razor blade under sterile conditions. The minced tissue was then digested in a solution of type II collagenase (prepared in sterile PBS, 5000U/heart, Worthington, 46H16739) for 45 min at 37°C with gentle agitation. Collagenase activity was then quenched with culture medium [i.e., Dulbecco’s Modified Eagle’s Medium/F12 GlutaMax (DMEM, Gibco 10565018) containing 10% fetal bovine serum (FBS, VWR 76324–888), 1% penicillin/streptomycin/amphotericin B (Sigma, A5955), 1% insulin transferrin selenium (ITS-G, Gibco 41400045), and 20 ng/ml human bFGF (Peprotech 100–18B)] and the mixture was then centrifuged at 500g for 10 min. Supernatants were aspirated, and cell pellets were resuspended in culture media before being passed through a 70 μm cell strainer. The cells were then plated in a T-75 cell culture flask (10062–860, VWR) and incubated at 37°C in 5% CO_2_. Media was changed after 2 h and again after 24 h. For *in vitro* studies, fibroblasts were used at passage 1 and serum-starved in DMEM/F12 GlutaMax media (ThermoFisher) without any supplementation prior to treatment with TGFβ (10 ng/ml, PeproTech, 100–21), bFGF (20 ng/ml, PeproTech, 100–18C), IL-1β (15 ng/ml, PeproTech, 200–01B), or TNF-α (10ng/ml, PeproTech, 300–01A). When appropriate, TAK1 inactivation in isolated cardiac fibroblasts from TAK1^fl/fl^ mice was induced prior to ligand stimulation with ad-CMV-iCre (Vector Biolabs, 1045) or vector control ad-CMV-LacZ (Vector Biolabs, 1080). Following 48 hr of virus transduction, cells were given a 48 hr rest period prior to experimental treatments.

### SUnSET assay:

Following experimental treatment, 1μM of puromycin (Millipore, P8833) was added to the cell culture medium for 30 mins prior to conditioned medium collection. Newly synthesized and secreted puromycin-labeled proteins were detected through immunoblot visualization using primary antibody anti-puromycin (1:10,000, Millipore, MABE343).

### Experimental animals and diets:

Experimental protocols were reviewed and approved by the University of Louisville Institutional Animal Care and Use Committee, and all animal studies were completed in compliance with the *Guide for the Care and Use of Laboratory Animals*. A fibroblast-specific TAK1 knockdown model was developed by crossing *Col1a2-Cre*^*ERT*+/−^ mice (Jackson Laboratory) with TAK1^fl/fl^ mice. All mice were on a C57BL/6J background and genotype was determined by tail DNA PCR. Animals were housed in a pathogen-free facility under controlled conditions (24°C, 44–65% relative humidity, 12-hour light/dark cycle) and maintained on standard chow diet purchased from LabDiet (Cat: 5010). For inactivation of TAK1, mice where either given i.p. injections of tamoxifen (75 mg/kg body weight, Sigma-Aldrich, T5648) for 5 days followed by a 5-day washout period or fed a chow containing tamoxifen (~400 mg/kg diet, Envigo, TD.130859) for 2 weeks followed by a 1-week washout period prior to experimental intervention.

### Non-reperfused myocardial infarction:

In a dedicated laboratory space, adult, 12-week-old, male and female Cre^+^/TAK1^fl/fl^ and TAK1^fl/fl^ littermate mice were acclimated for at least 1 h prior to non-reperfused myocardial infarction, as described. ^34,35^ Briefly, mice were first anesthetized through intra-peritoneal injections of (pentobarbital, 50 mg/kg; ketamine, 50 mg/kg) before surgery. Subsequently, they were orally intubated and ventilated with oxygen. Using a 7–0 silk suture, the left coronary artery was permanently ligated and then the chest wall was sutured closed. Mice were extubated only after the recovery of spontaneous breathing, and analgesia (meloxicam, 20 mg/kg) was provided prior to the recovery as well as once a day at 24 and 48 h post-surgery. The surgeon was blinded to mouse genotype. Echocardiography confirmation of infarction was required for inclusion in subsequent analysis.

### Transverse Aortic Constriction:

In a dedicated laboratory space, adult, 12-week-old, male and female Cre+/TAK1^fl/fl^ and TAK1^fl/fl^ littermate mice were acclimated for at least 1 h prior to transverse aortic constriction surgery, as described.^36–38^ Briefly, mice were anesthetized with 50 mg/kg ketamine + 50 mg/kg pentobarbital and maintained with 1% isoflurane. Chords were visualized, and mice orally intubated using PE-60 tubing and maintained under anesthesia with an isoflurane vaporizer supplemented with oxygen. A skin incision was made left of the midline skin to expose the chest. Using a cautery tip, a small incision was made between the 3^rd^ and 4th rib on the left side of the chest. A 7–0 silk suture was then looped around the aorta between the brachiocephalic and left common carotid arteries. The suture was tied around a 27G needle to occlude the aorta. The needle was quickly removed, and constriction was visualized before the chest was closed with a 4–0 monofilament suture. Mice were extubated upon recovery of spontaneous breathing and allowed to recover in a warm clean cage. Analgesia (meloxicam, 20 mg/kg) was provided prior to the recovery as well as once a day at 24 and 48 h post-surgery. The surgeon was blinded to mouse genotype. Only mice with a trans-constriction pressure gradient of ≥40 mmHg were considered for subsequent analysis.

### Post-operation Animal Care:

Research team and veterinary staff monitored the animals twice daily during the 1^st^ week after surgery and then twice weekly throughout the duration of the study. Health was gauged by temperature, body weight, food and water intake, and general assessment of respiratory status, ambulation, arousal, posture and surgical site healing. Indications for euthanasia include loss of body weight (≥15%), respiratory stress, suture site dehiscence, hunched posture, surgical site infections, vocalization to touch, or other clinical signs of distress as listed in the University of Louisville Institutional Animal Care and Use Committee Humane Endpoints Policy.

### Echocardiography Acquisition and Analysis:

Cardiac structure and function were assessed through transthoracic echocardiography (Vevo 3100, Visual Sonics) while under anesthetic (isoflurane) at the designated times. Ventricular wall thickness was obtained with M-mode images through parasternal long axis (PLAX) view. Reported measurements included left ventricular (LV) interior diameter (LVID), LV mass (corrected), LV posterior wall thickness, and LV anterior wall thickness near the border zone. Each parameter was the cumulative average of four measurements and LV mass (corrected) was calculated from the formula: (1.053 × [(LV interior diameter;d + LV posterior wall;d + intravenous septum;d)^3^ – LV interior diameter;d^3^]) × 0.8 (d = diastole). Additionally, for uninjured and TAC hearts, two-dimensional images viewed in B-Mode from the parasternal long axis (PLAX) was used to make measurements for left ventricular end-diastolic volume (LVEDV), left ventricular end-systolic volume (LVESV), stroke volume (SV), cardiac output (CO), ejection fraction (EF), and heart rate (HR). Six measurements were taken and averaged for final outputs. For myocardial infarction studies, Simpson’s method B-mode with PLAX and apical view was used to obtain images for measuring volumetric (LVEDV, LVESV) and cardiac performance (SV, CO, EF, HR) indices with five measurements taken at three different ventricular planes and averaged. An apical four chamber view in Pulsed Wave (PW) Doppler was used for measurement of isovolumic relaxation time (IVRT) for all studies, reported values were averaged from nine individual measurements. The sonographer and analyzer were blinded to mouse genotype.

### Histopathology and scar quantification:

At the conclusion of the study, hearts were excised and arrested in diastole with ice-cold 2% potassium chloride. Each heart was then sectioned into 2-mm cross-sectional segments and fixed in 10% formalin prior to being embedded in paraffin, sectioned (4 μm), and mounted. Slides were then deparaffinized and rehydrated before staining. Picrosirius red staining (Picric Acid, Sigma, P6744; Direct Red 80, Sigma, 365548) was used to detect collagen in intact sections from base to apex. Sections were visualized using the Keyence Imaging System. Acquired images were then analyzed using the Keyence BZ-X800 analyzer. The microscopist was blinded to group assignments. Furthermore, total scar size (mass in mg) was calculated by summation of scar masses corresponding to each transverse myocardial segment (base to apex; 4 segments per heart): scar mass (mg) = [section mass (mg)] × [scar area (μm^2^)/section area (μm^2^)]. Infarct width was manually measured via ImageJ in midpapillary level sections.

### Western Blot Analysis:

Following experimental treatments, conditioned medium was collected and total protein was harvested after washing cells with ice-cold PBS. PBS was aspirated and cells were then scraped with a rubber policeman in lysis buffer (20 mM HEPES, 110 mM KCl, 1 mM EDTA, 1% IGE-PAL, and 0.1% SDS, pH 7.0) containing protease inhibitor cocktail (Sigma-Aldrich, P8340). The cell lysates were incubated on ice for 30 min and then centrifuged at 20,000*g* for 20 min. The supernatants were then carefully removed and saved for total protein measurements (Lowry assay) and Western blotting.

Conditioned medium and cell lysate protein samples were mixed with Laemmli sample buffer (125 mM Tris-HCl, 10% SDS, 50% Glycerol, 0.05% Bromophenol Blue, pH 6.8) and incubated at 95°C for 5 min. Each sample was then loaded on a 7.5% acrylamide/bis SDS-PAGE gel for electrophoresis at 120 V. SDS-PAGE-resolved proteins were then transferred overnight onto a PVDF membrane (Cytiva, 10600021) at 4°C at 20 V for 16 h. Subsequently, membranes were blocked for 1 h at room temperature with Tris-buffered saline containing Tween-20 (TBS-T) and 5% bovine serum albumin (Sigma-Aldrich, A7906). Membranes were probed with primary antibodies overnight at 4°C. The following day, membranes were washed three times using TBS-T and then incubated with secondary antibody for 1.5 h. Membranes were then washed three times with TBS-T before exposure to Pierce^™^ ECL Plus Western Blotting Substrate (Thermo Scientific, 32132) and imaging on a ChemiDoc imager (BioRad). Protein was normalized to total lane protein with an amido black stain, and parallel blots were normalized to an anchor protein. The primary antibodies used include: anti-COL1A1 (1:20,000; Thermo Fisher, PA5–29569), anti-Periostin (1:5,000; Millipore, ABT253), anti-α-smooth muscle actin (1:30,000; Cell Signaling, 19245S), anti-MMP3 (1:2,000, Invitrogen, MA5–42477), anti-COX2 (1:2,000, Cell Signaling, 12282), anti-iNOS (1:2,000, Millipore, 06–573), anti-TAK1 (1:500, Cell Signaling, 5206), anti-p-NFκB p65 (1:1,000) Cell Signaling, 3033), anti-NFκB p65 (1:1,000, Cell Signaling, 8242), anti-p-SAPK/JNK (1:1,000, Cell Signaling, 4668), anti-SAPK/JNK (1:1000, Cell Signaling, 9252), anti-p-p38 (1:5,000, Cell Signaling, 4511), anti-p38 (1:5,000, Cell Signaling, 8690), anti-IκBα (1:2,000, Cell Signaling, 9242), anti-p-Smad3 (1:2,000, Cell Signaling, 9520), anti-Smad3 (1:2,000, Cell Signaling, 9523T). Secondary antibody included anti-rabbit IgG HRP-linked (1:2,500; Cell Signaling, 7074) and anti-mouse IgG HRP-linked (1:2,500; Cell Signaling, 7076).

### Flow Cytometry:

Post-MI hearts were harvested, and PBS was perfused through the aorta for 5 min. Following, the aorta, atria, and excess adipose was excised from the ventricles. The tissue was then finely minced with a razor blade and digested in PBS with collagenase II (1200 U/mL, Worthington Biochemical) with gentle agitation for 45 min at 37°C. Collagenase was deactivated by washing with PBS and cells were further separated using a 30% Percoll gradient (Sigma Aldrich, P4937). After centrifugation, debris was aspirated, and cell pellets were washed with PBS. Cells were stained with antibodies for 30 min at 4°C in the dark: APC anti-mouse Ly6C (Biolegend, 128016), BV 421 anti-mouse CD192 (Biolegend, 150605), PE anti-mouse CD64 (Biolegend, 139304), PE-Cyanine7 monoclonal antibody CD45 (Thermo Fisher, 25–0451-82), FITC monoclonal antibody MHC class II I-Ab (Thermo Fisher, 11–5320-82). Finally, cells were washed twice with PBS and resuspended in 1 mL of PBS for flow cytometry. Immune cells were first gated as CD45^pos^ followed by monocytes as CD64^pos^CCR2^pos^MHCII^lo^ and neutrophils through an exclusion criteria^18^. Monocyte-derived macrophages were gated as CD64^pos^CCR2^pos^MHCII^pos^ while resident macrophages were defined as CD64^pos^CCR2^neg^MHCII^pos^. Subsequent data analysis was completed using Flowjo, LLC.

### Hyaluronan Isolation, Sizing, and Analysis:

Hyaluronan was isolated as described^39,40^, with slight modifications. For cultured fibroblast media, hyaluronan (HA) was isolated from conditioned media of treated fibroblasts. Proteins were digested using 1´ proteinase K (Invitrogen, 25530015) at 60°C for 4 h and first precipitation was done with 200 proof ethanol at −20°C (Day 1). Samples are then washed with 75% ethanol, resuspended in 100 mM ammonium acetate (Sigma Aldrich, A1542) and nucleic acids were digested with Benzonase (EMD Millipore, 70664–3 25 U/μl) at 37°C (Day 2). Second precipitation was done with 200 proof ethanol and samples were washed with 75% ethanol. Samples were air-dried, resuspended in 100 mM ammonium acetate (Sigma Aldrich, A1542) and incubated at 4°C (Day 3). Samples were lyophilized and resuspended in 10 M formamide (Sigma Aldrich, F9037) and incubated at 4°C (Day 4). A 1% agarose gel was made with 1´ TAE was used to size HA. After casting, gel was pre-run in 1´ TAE for 6 h at 80 V to remove impurities. Loading buffer (0.2% bromophenol blue (Sigma Aldrich, B0126) in 10 M formamide) was added to each sample and HA^HMW^ (R&D Systems, GLR002) positive control. Samples were run on gel in 1´ TAE at 100 V for 2.5–3.0 h and equilibrated in 30% ethanol for 1 hour before being stained with 1 mL of 400´ stock stains-all solution (12.5 mg of stains-all (Sigma Aldrich, E9379) to 5 mL of 200 proof ethanol) in 400 mL of 30% ethanol in the dark, overnight (Day 5). Gels were equilibrated in dd-water for 1 h in the dark and destained under ambient light on the bench for 15–20 min. Color images were taken on an EPSON flatbed scanner for presentation purposes. Image for quantitation was taken using a Bio-Rad ChemiDoc using the Stain-Free setting. Stain-Free images were analyzed using Fiji-Image J.

### Multiplex analysis, MD44:

To profile secreted proteins in the conditioned medium, we used Luminex^®^ xMAP^®^ technology to quantitatively and simultaneously detect forty-four mouse cytokines, chemokines and growth factors. The multiplexing analysis was performed by Eve Technologies Corporation (Calgary, Alberta, Canada) using the Luminex^®^ 200^™^ system (Luminex Corporation/DiaSorin, Saluggia, Italy) with Bio-Plex Manager^™^ software (Bio-Rad Laboratories Inc., Hercules, California, United USA). Forty-four markers were measured in the samples using two separate Eve Technologies’ panels, as per the manufacturer’s instructions for use: Mouse Cytokine/Chemokine 32-Plex Discovery Assay^®^ Array (MD32) (MILLIPLEX^®^ Mouse Cytokine/Chemokine Magnetic Bead Panel Cat. #MCYTOMAG-70K, MilliporeSigma, Burlington, Massachusetts, USA) and Mouse Cytokine/Chemokine 12-Plex Discovery Assay^®^ Array (MD12) (MILLIPLEX^®^ Mouse Cytokine/Chemokine Magnetic Bead Panel II Cat. # MECY2MAG-73K, MilliporeSigma, Burlington, Massachusetts, USA). Assay sensitivities of these markers range from 0.3 – 30.6 pg/mL. Individual analyte sensitivity values are available in the MilliporeSigma MILLIPLEX^®^ protocols.

### Lipid mediator quantification by targeted liquid chromatography-tandem mass spectrometry (LC-MS/MS): Lipid mediator quantification by targeted liquid chromatography-tandem mass spectrometry (LC-MS/MS):

To quantify lipid mediators, 6 mL of culture media collected from 6×10^5^ cardiac fibroblast was immediately frozen following IL-1β treatment and stored at −80°C. Before performing solid phase extraction, samples were thawed and concentrated to 300 μL under a steady stream of N_2_ gas at 37°C using a Turbovap (Biotage, Uppsala, Sweden) and stored at −80°C following the addition of 600 μl of HPLC-grade MeOH. On the day of extraction, 100 μL of HPLC-grade MeOH containing 500 pg of deuterium-labeled internal standards (i.e. Resolvin D_2_-d_5_, Resolvin D_3_-d_5_, Maresin 1-d_5_, Maresin 2-d_5_, Lipoxin A_4_-d_5_, Resolvin E_1_-d_4_, 5(S)-HETE-d_8_, 15(S)-HETE-d_8_, (+)11(12)-EET-d_11_, 15-deoxy-Δ12,14-Prostaglandin J_2_-d_4_, Prostaglandin E_2_-d_4_, and Leukotriene B_4_-d_4_) were added to calculate extraction efficiency. Protein precipitates were pelleted by centrifuging samples at 6,200 RCF for 10 min at 4°C and supernatants were transferred into 12 mL borosilicate glass round-bottom tubes and concentrated to 200 μL total volume. Samples were acidified by the addition of pH 3.5 HPLC-grade H_2_O. Using an Extrahera automated extraction system (Biotage, Uppsala, Sweden), samples were promptly loaded onto Biotage ISOLUTE C18 SPE columns (cat. no. 220–0020-c, Biotage, Uppsala, Sweden), which had been pre-equilibrated with 3 mL of HPLC-grade MeOH and 3 mL HPLC-grade H_2_O. After samples were loaded, columns were then neutralized with 5 mL of HPLC-grade H_2_O and subsequently treated with 5 mL of HPLC-grade hexane to remove neutral lipids. Lipid mediators of interest were then eluted and collected by the addition of 5 mL HPLC-grade methyl formate. Samples were dried to completion with a gentle stream of N_2_ gas using a Biotage TurboVap Classic and immediately resuspended in 50 μL of HPLC-grade MeOH:H_2_O (50:50 v/v). LC-MS/MS analysis was carried out on samples using a Kinetex Polar C18 HPLC column (100 mm length x 3 mm diameter; 2.6 μm particle size; 100 Å pore size) (cat. no. 00D-4759-Y0, Phenomenex, Torrance, CA, USA) maintained at 59.0°C using a Shimadzu LC-20AD with a SIL-20AC autoinjector (Shimadzu, Kyoto, JP), coupled to a 5500 Qtrap (Sciex, Toronto, ON, Canada) as described before^41^. Targeted LC-MS/MS analysis was carried out first by gradient elution of lipid mediators with a constant 0.5 mL/min mobile phase delivery, initially consisting of 45:55:0.01 (v/v/v) methanol:water:acetic acid, which was then ramped up to 80:20:0.01 over 16.5 min, and then to 98:2:0.01 over the next 2 min. The 5500 Qtrap was operated in negative polarity mode. All data were acquired using Analyst v1.7.1 and analyzed with Sciex OS-Q v3.0.0.3339 software (Sciex). Each analyte was identified using multiple reaction monitoring (MRM) and enhanced product ion (EPI) scans (Supplemental Tables S2-S4) with retention time (RT) matching to synthetic standards (Fig. 2F). A given chromatographic peak was quantified if the following criteria were met: (1) RT was within ± 0.1 min of the synthetic standard’s RT; (2) chromatographic peak was composed of at least 5 points across baseline; (3) MS/MS spectra at the appropriate RT contained at least 6 diagnostic ions; (4) signal-to-noise ratio (SNR) was greater than 3; (5) the calculated analyte concentration was greater than the empirically-determined LLOD and LLOQ for the respective analyte. Sciex OS-Q Autopeak Integration Algorithm was used to integrate peaks and to generate peak area and SNR values.

Determination of the lower limit of detection (LLOD) and lower limit of quantitation (LLOQ) was carried out as recommended by the International Council for Harmonization analytical guideline Q2 (R2) (https://www.ich.org/page/quality-guidelines). Briefly, for all analytes, calibration standards (Cayman Chemical Company, Ann Arbor, MI, USA) were prepared by mixing twelve synthetic standards with a beginning concentration of 100 pg/μl (Standard 1) and serially diluted in HPLC-grade methanol (Sigma-Aldrich) by 1:2 dilution to a final concentration of 0.048 pg/μl (Standard 12). For each standard, an injection volume of 5 μl was used. At least 5 independent injections of each standard were used to generate data to determine LLOD and LLOQ for each analyte. From the accumulated data, the slope (S) of the calibration curve and the standard deviation (σ) of the y-intercept, which represents the blank condition, were calculated. LLOD and LLOQ were calculated using 3.3*σ/S and 10*σ/S, respectively.

An MS/MS spectra matching library was generated using Sciex OS LibraryView v1.4 (Sciex). To generate this library, 5 μl of synthetic standards at concentrations of 100pg/μl, 6.25pg/μl and 0.195pg/μl were injected to create a range of concentration-based MS/MS reference spectra that were added to the LibraryView database. This lipid spectra library was selected as part of the Sciex OS Process Method Library Search function. The search utilized the Smart Confirmation Search and Fit sorting options. The algorithm parameters Precursor Mass Tolerance and fragment Mass Tolerance were both set to ± 0.7Da. The Use Polarity Intensity Threshold option was set to 0.03. For each chromatographic peak quantified, a library matching score was generated and used to confirm the proper identification of the analyte based on reference MS/MS spectra.

To determine pg of analyte in each sample, first, a 12-point calibration curve was created for each analyte in the standard mix by using a ratiometric method. With this method, a fixed (500) pg amount of internal deuterated standard (IS) was mixed with decreasing amounts of each synthetic standard, namely – 500, 250, 125, 62.5, 31.25, 15.63, 7.81, 3.91, 1.95, 0.98, 0.49, or 0.24 pg. The ratio of the peak areas was calculated and used as the Y-axis value, while the ratio of known pg of IS to synthetic standard on each calibration curve sample was used as the X-axis value to generate a calibration curve. All biological samples were spiked with the same pg of deuterated internal standards before extraction, and the peak area ratios of IS and analytes were calculated, and subsequently fitted into the calibration curve using a weighted (1/x) linear regression method to obtain pg of analyte in the biological sample. Each pg of analyte value was normalized to volume of starting cell culture media.

### RNA sequencing:

Total RNA was isolated from murine cardiac fibroblast using the Qiagen RNeasy Micro Kit (74004). RNA quality was evaluated using NanoDrop ONEC (Thermo Scientific), and 100 ng of RNA was sent to sequencing technology center for poly-A RNA sequencing. The TruSeq Stranded mRNA Library Prep Kit (Illumina Cat# 20020594) and TruSeq RNA CD Index Plate (Illumina Cat# 20019792) were used to generate a sequencing library. Size, purity, and semi-quantitation were performed on an Agilent Bioanalyzer 2100 system using the Agilent DNA HS Kit. Single-end sequencing was performed on an Illumina NextSeq 2000, using a P3 100 cycle cartridge with a P3 flow cell. Libraries were sequenced with a single 101 cycle read length with 2 index reads of 8bp each. FASTQ files were generated utilizing BaseSpace DRAGEN analysis Version 1.2.1.

### Bioinformatics Analysis:

Quality control (QC) of the raw sequence data was performed using FastQC (version 0.10.0) for each sequencing sample^42^. The sequences were aligned to the mm10 mouse reference genome using STAR version 2.6.^43^ Differential expression of ENSEMBL protein-coding transcripts was performed using DESeq2^44^, and raw counts were obtained from the STAR-aligned bam format files using HTSeq version 0.10.0^45^. The raw counts were normalized using the Relative Log Expression (RLE) method and then filtered to exclude genes with fewer than ten counts across the samples. DESeq2 guidelines were used to identify differentially expressed genes, and all P values were adjusted for testing multiple genes (Benjamini–Hochberg procedure; p≤0.05). RNA-seq data were deposited in the NCBI GEO database under Accession No. GSE290127

### Statistical Analysis:

Data are mean ± SEM. Normality was assessed via Shapiro-Wilk test were appropriate. Statistical analyses were performed using log-rank Mantel Cox test, parametric unpaired student’s t-test, non-parametric Mann-Whitney U test, ordinary one-way ANOVA with Dunnett post-hoc test, or two-way ANOVA with Šidák’s post-hoc test, as appropriate. The null hypothesis was rejected when p < 0.05. All statistical analyses were performed using GraphPad Prism, version 10 (GraphPad Software, La Jolla, California).
